# Postsynaptic protein organization revealed by electron microscopy

**DOI:** 10.1016/j.sbi.2019.02.012

**Published:** 2019-03-21

**Authors:** Yun-Tao Liu, Chang-Lu Tao, Pak-Ming Lau, Z Hong Zhou, Guo-Qiang Bi

**Affiliations:** 1 Center for Integrative Imaging, Hefei National Laboratory for Physical Sciences at the Microscale, University of Science and Technology of China, Hefei, Anhui, China; 2 School of Life Sciences, University of Science and Technology of China, Hefei, Anhui, China; 3 The California NanoSystems Institute, University of California, Los Angeles, CA, USA; 4 Department of Microbiology, Immunology and Molecular Genetics, University of California, Los Angeles, CA, USA; 5 CAS Center for Excellence in Brain Science and Intelligence Technology, University of Science and Technology of China, Hefei, Anhui, China

## Abstract

Neuronal synapses are key devices for transmitting and processing information in the nervous system. Synaptic plasticity, generally regarded as the cellular basis of learning and memory, involves changes of subcellular structures that take place at the nanoscale. High-resolution imaging methods, especially electron microscopy (EM), have allowed for quantitative analysis of such nanoscale structures in different types of synapses. In particular, the semi-ordered organization of neurotransmitter receptors and their interacting scaffolds in the postsynaptic density have been characterized for both excitatory and inhibitory synapses by studies using various EM techniques such as immuno-EM, electron tomography of high-pressure freezing and freeze-substituted samples, and cryo electron tomography. These techniques, in combination with new correlative approaches, will further facilitate our understanding of the molecular organization underlying diverse functions of neuronal synapses.

## Introduction

Neuronal synapses are basic functional units in the brain playing essential roles in information transduction, processing, and storage [1]. Inside a synapse, numerous protein molecules organize in a partially ordered manner, forming specialized synaptic architecture to carry out signaling and structural tasks [2,3•,^4^]. In the past decades, much has been learned about the identity of synaptic proteins and the topology of their interactions based on molecular and biochemical studies [5]. Knowledge regarding the fine structure of synapses has been obtained primarily through studies using electron microscopy (EM) [6,7,8••,9•,^10^–^12^].

In this article, we review recent progress in our understanding of synaptic ultrastructure and molecular organization mainly based on electron microscopic analyses. We focus on the molecular organization of the postsynaptic compartment of mammalian central nervous system synapses, especially neurotransmitter receptors and scaffolding proteins in the postsynaptic densities (PSDs). We also discuss current and future applications of other tools such as correlative light and electron microscopy (CLEM) in studying specific types and functional states of synapses. The presynaptic architecture and organization of synaptic vesicles are topics of another review in this issue.

## Electron microscopy techniques used in the study of neuronal synapses

Three types of EM-based techniques have been used to study the organization of proteins inside synapses: immuno-EM, electron tomography (ET) of high-pressure freezing and freeze substituted (HPF-FS) samples, and cryo electron tomography (cryoET).

Immuno-EM uses antibodies conjugated with electron-dense gold particles to directly or indirectly label specific proteins [13]. The high spatial precision (~10 nm) of EM and high specificity of the antibody-antigen interaction allows for localization of individual protein molecules, for example, different subtypes of a-amino-3-hydroxy-5-methyl-4-isoxazolepropionic acid receptors (AMPAR) on the postsynaptic membrane [9•, 14,15]. However, the label density of immuno-EM is often low and varies depending on sample preparation conditions such as pre-embedding or post-embedding immuno-gold labeling. Combining with SDS-digested freeze-fracture replica labeling (SDS-FRL), immuno-EM has been used to reveal the organization of membrane proteins. With SDS-FRL, a large fraction of the membrane proteins can be sufficiently exposed to immunogold labeling. The number and distributions of AMPARs or g-aminobutyric acid type A receptors (GABA_A_Rs) on postsynaptic membranes have been systematically analyzed with this method [7,16,17].

High-pressure freezing followed by freeze-substitution significantly improves the preservation of synaptic ultrastructure, avoiding distortions of cellular morphology induced by chemical fixation [18]. By combining HPF-FS sample preparation with electron tomography (ET), which obtains high-resolution 3D ultrastructure by reconstruction from projections at different angles of the sample [19], postsynaptic glutamate receptors and MAGUK family proteins have been visually identified and localized according to their shapes [20,21••].

**Figure 1:**
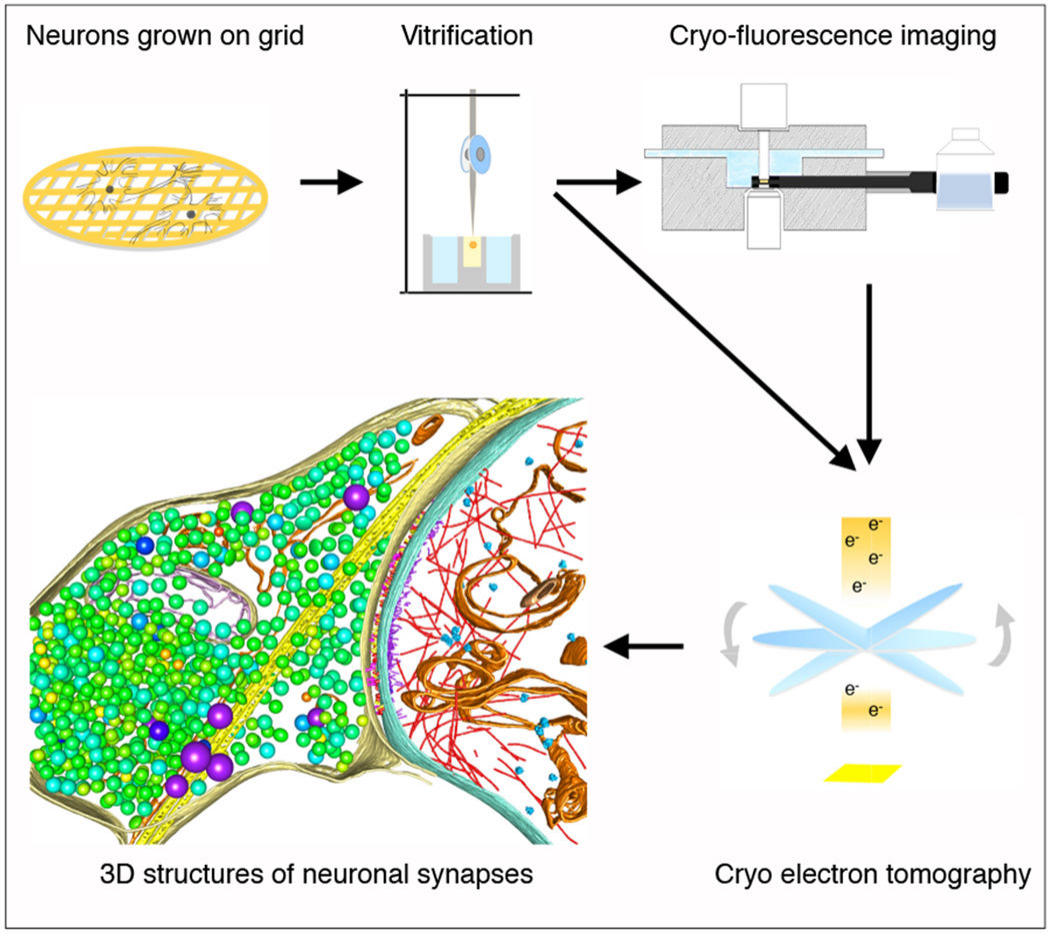
Visualizing synaptic ultrastructure in cultured neurons with cryoET and correlative microscopy. Primary neurons dissociated from the brain are cultured on gold EM grids, and are directly cryo-fixed with plunge freezing. The vitrified samples are imaged with cryoET or in combination with correlative light microscopy. Electron micrographs collected for a series of imaging tilt angles are processed for reconstruction and segmentation, resulting in 3D ultrastructure of the synapse. Adapted from Ref. [8••].

**Figure 2:**
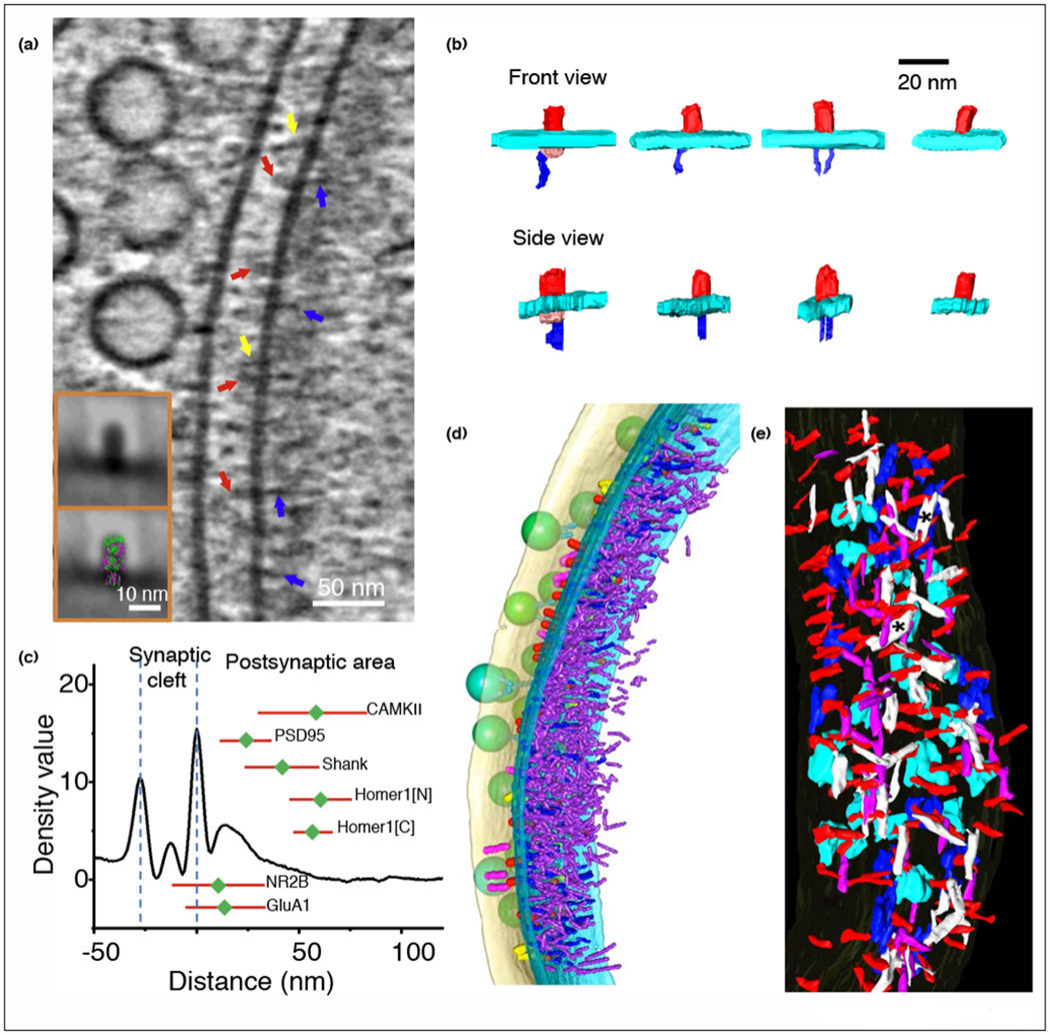
Putative receptors and scaffolding proteins in excitatory synapse. **(a)** An 8.7 nm thick tomographic slice of an excitatory synapse around the synaptic cleft region. Arrows point to proteins on the postsynaptic membrane: putative receptors (red), putative adhesion molecules (yellow), and PSD filaments (blue). Inset are averaged images of putative glutamate receptor without and with atomic structures of AMPAR (green) [72] and NMDAR (pink) [73] superposed. **(b)** Examples of segmented putative glutamate receptor densities (red) with their interacting PSD molecules (blue). **(c)** Averaged density profile perpendicular to postsynaptic membrane of excitatory synapses. Green dots and red bars indicate axial positions of different synaptic proteins as measured using super-resolution light microscopy (mean: green dots. SD: red bar, data from Ref. [49]). **(d)** 3D segmentation from the cryoET tomogram of the excitatory synapse as in (a), showing the organization of scaffolding proteins attached to the membrane (blue) and deeper into the PSD (purple). **(e)** Organization of the PSD core in an excitatory synapse from HPF-FS ET, showing postsynaptic proteins including cytoplasmic domains of AMAPRs (blue) and NMDARs (cyan), as well as vertical (red) and horizontal (white and magenta) filaments. Figure 2a–d was adapted from Ref. [8••]. Figure 2e was adapted from Ref. [21••] Copyright (2008) National Academy of Sciences.

**Figure 3:**
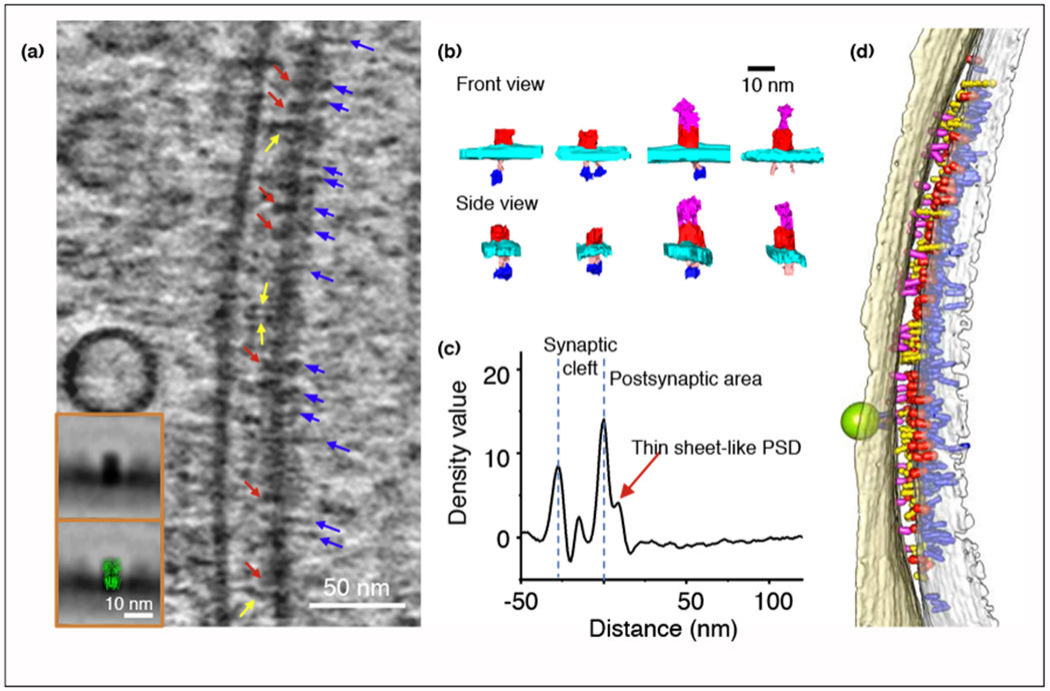
Putative receptors and scaffolding proteins in inhibitory synapse. **(a)** An 8.7 nm thick tomographic slice of an inhibitory synapse around the synaptic cleft region. Arrows point to proteins on the postsynaptic membrane: putative receptors (red), putative adhesion molecules (yellow), and PSD filaments (blue). Inset are averaged images of putative glutamate receptor without and with atomic structure of GABA_A_R (green) [74] superposed. **(b)** Examples of segmented putative GABA_A_R and interacting scaffolds, including extracellular densities of receptors (red), as well as intracellular neck densities (pink) and head densities (blue) of the receptor/scaffold complex. **(c)** Averaged density profile perpendicular to postsynaptic membrane of inhibitory synapses. **(d)** 3D segmentation from the cryoET tomogram showing putative receptors (red), postsynaptic filaments (blue) on the postsynaptic membrane, and adhesion molecules (yellow and magenta) in the synaptic cleft. Adapted from Ref. [8••].

The advent of cryo electron microscopy techniques, especially with the recently developed direct electron detector, has made it possible to resolve the structure of proteins at atomic resolutions [22]. CryoET is the tool of choice to observe the 3D structure of pleomorphic samples such as neuronal synapses *in situ* [23,24]. The advantage of cryoET is that the samples are not dehydrated, so that synapses and other cellular structures can be preserved in their native state (Figure 1), with protein complexes observed based on their intrinsic density rather than the shade of heavy metal staining as in classical EM [23,24]. In early applications, cryoET was used to resolve presynaptic vesicular organizations and synaptic clefts in isolated synaptosomes [25,26]. In our recent studies, cryoET was used to visualize the 3D ultrastructural features of excitatory and inhibitory synapses (Figures 2 and 3), and to analyze changes in the distribution of presynaptic dense core vesicles under chronic inactivation in intact cultured hippocampal neurons [8••,^27^]. The application of Volta phase plate and electron energy filter has further improved the contrast and resolution of cryoET reconstructions, enabling the identification of neurotransmitter receptors (Figures 2 and 3) [8••].

## Organization of neurotransmitter receptors in excitatory synapses

The number and organization of neurotransmitter receptors on the postsynaptic membrane are important determinants of synaptic efficacy [15,28]. ET imaging of HPF-FS samples provided the first 3D map of AMPARs and *N*-methyl-d-aspartate receptors (NMDARs) in the postsynaptic membrane based on visually segmented shapes [21••]. With HPF-FS ET, glutamate receptor-like particles were observed to have extracellular structures of ~15 nm long, ~8nm wide, and 9 nm high, consistent with the sizes of atomic-resolution structures of the extracellular domains of AMPAR and NMDAR [21••]. Remarkably, AMPAR contains a small flat cytoplasmic domain whereas NMDAR contains a large globular cytoplasmic domain of about 20 nm [21••], which could be essential for triggering intracellular signaling cascade and forming supercomplexes [29]. With cryoET, AMPARs, and NMDARs were also identified on the postsynaptic membrane based on visual classification (Figure 2a and b) [8••]. However, under cryoET, the cytoplasmic domains of NMDARs appeared to be substantially smaller than those observed with HPF-FS ET. It is possible that the large size of NMDAR cytoplasmic domains with HPF-FS ET was partially due to heavy metal staining of the receptors together with their intracellular binding partners.

Using immuno-EM method, Kharazia and Weinberg found that AMPARs were generally located at the periphery of postsynaptic membranes, whereas NMDARs were more centrally located [14]. This ‘center-surround’ preferential distribution of the two receptors was also confirmed by subsequent HPF-FS ET study (Figure 2e) [21••], and is consistent with the idea that AMPARs are rapidly recruited to postsynaptic membrane from the extra-synaptic area during long-term potentiation [30,31]. Recent studies using super resolution optical microscopy observed that AMPARs form clusters of ~80 nm in size called nano-domains [32,33]. Such center-surround [14,21••], nano-domain [32,33] and even random [7,17] organizations are statistically significant ensemble properties, but not necessarily prominent features for given individual synapses. It is possible that the exact form of receptor organization depends on the type and plasticity state of the synapse. Indeed retinogeniculate and corticogeniculate synapses were found to prefer uniform and clustered AMPAR distributions, respectively [34•].

Further study with super-resolution optical microscopy shows that some of the nano-domains of AMPARs are aligned with nano-domains of Rab3-interacting molecules (RIM), a key component of the presynaptic vesicle releasing machinery, to form ‘nano-columns’ [3•,^35^]. This trans-synaptic alignment could provide a mechanism for efficient synaptic transmission [3•,^32^,^33^,34•]. On the other hand, such alignment between clustered receptors and presynaptic vesicle release machinery has not been documented in EM studies. Intriguingly, trans-cleft molecules in both excitatory and inhibitory synapses as observed by HPF-FS ET appear to avoid the space corresponding to presynaptic vesicle docking sites [36]. In contrast, super-resolution optical imaging suggests that cell adhesion molecules in the synaptic cleft such as neuroligin-1 link AMPAR clusters with presynaptic RIM [37]. These discrepancies may reflect structural heterogeneity of different types and states of synapses, and could be addressed by future studies with high-resolution approaches such as cryoET and correlative microscopy that can directly analyze the organization of individual synaptic protein molecules.

## Organization of postsynaptic scaffolding proteins in excitatory synapses

Underneath the postsynaptic membrane lies a specialized structure called the postsynaptic density, which contains high levels of synaptic proteins of various functions, including scaffolding, cytoskeletal, and signaling proteins [5]. These proteins are strongly stained with heavy metals, making the PSD a hallmark of the excitatory synapse under conventional EM [38,39]. From cryoET images of native synaptic structure, density profile of the PSD measured perpendicular to the postsynaptic membrane showed a peak at about 15 nm from the membrane, and a long tail extending to ~50 nm away (Figure 2c) [8••]. This is consistent with the idea of laminar organization of the PSD: the PSD consists of a dense core of PSD proteins near the membrane and a less dense ‘pallium’ of ~50 nm thickness [9•,^40^].

Biochemical studies show that MAGUK family proteins such as PSD-95 can directly bind to GluN2 (a subunit of NMDAR) [41] and to stargazin (an auxiliary regulatory protein for AMPAR) [42]. Using HPF-FS ET, Chen *et al.* showed that PSD-95 anchors with its N-terminus on the cytosolic sides of these receptors and projects ‘vertically’ into the cytoplasm, that is, perpendicular to the postsynaptic membrane (Figure 2e) [21••]. Using cryoET, similar vertical filaments can also be observed (Figure 2d) [8••]. Most of these filaments are straight but of different lengths, with some filaments containing a kink (Figure 2b), suggesting that they may represent different proteins or complexes. Interestingly, the interaction between vertical filaments and receptors varies: a receptor may link to zero, one, or two filaments (Figure 2b) [8••]. This variability is likely to reflect the dynamic nature of the interaction among receptors and scaffolding protein. In particular, a significant portion of AMPARs (10 out of 54 analyzed in the tomogram of an excitatory synapse) was found to have no vertical filament attached to the cytoplasmic side, consistent with the observation of substantial diffusion behavior of AMPARs. Dynamic interaction among receptors and scaffolds could also allow these molecules to form clusters through self-organization, which might underlie the formation of receptor nano-domains [32,33] as well as the patchy loss of the vertical filaments after PSD-95 knock-down [20,43].

At the cytoplasmic end of PSD-95 vertical filaments, horizontal filaments that are parallel to the postsynaptic membrane were observed to form a lateral network (Figure 2e) [21••]. These filaments could link PSD core with PSD pallium, and might be proteins such as GKAP, which links MAGUK family proteins to Shank [44,45]. Major proteins lying in the PSD pallium include Shank and Homer [46,47], which form a meshwork upon negative staining EM analysis [48]. The relative location of some of the PSD proteins has been quantitatively measured using super resolution stochastic optical reconstruction microscopy (Figure 2c) [49], confirming the laminar structure of the PSD. It is worth noting that the PSD pallium is also a dynamic structure: upon intense synaptic activation by chemical stimulation, Ca^2+^/calmodulin-dependent protein kinase II (CaMKII) molecules are recruited to the PSD [50], resulting in thickening of the PSD [51]. Although the functional significance of this recruitment has not been directly tested, it may be of relevance for synaptic functions such as activity-dependent plasticity that underlies learning and memory functions of the brain [52].

Thus, the static electron micrograms (or tomograms) are only snapshots of a more dynamic picture, where interactions among PSD proteins in both the core and the pallium vary over time. If this is the case, why these protein molecules do not diffuse away, leading to disintegration of the PSD structure? One possibility is that the dynamics are highly regulated, such that the molecules can diffuse only after specialized activation. Alternatively, these PSD proteins may be in a special state similar to liquid-phase condensates formed through multivalent interactions [53,54]. Recent studies have shown that PSD proteins including SynGAP and PSD-95 when mixed together could undergo phase separation *in vitro*, forming condensates containing high concentration of multivalent protein complexes [55•,^56^]. In electron tomograms from HPF-FS ET and cryoET [8••,21••], the PSDs appear to be organized in a mixed state: receptors and the vertical filaments near the membrane, as well as some distinct horizontal filaments in the core layer, are organized in a semi-ordered manner (Figure 2d and e). Beyond this core layer, the interaction among filaments of various orientations becomes more chaotic. This is clear in cryoET images, where most of the volume of the PSD is occupied by rather uniform weak densities (Figure 2a). Moreover, this uniform PSD area has a clear boundary from the rest of cytosolic regions, characteristic of liquid phase separation [54]. We suspect that the distinct filaments form a relatively more stable (though not permanent) gel-like framework for other scaffolding and signaling molecules to cluster around, which by multivalent interactions may form a liquid phase condensate of PSD proteins similar to that formed *in vitro* [55•,^56^]. It would be interesting to examine how such condensate may change during synaptic activation and plasticity, for example, whether the aforementioned activity-dependent recruitment of CaMKII effectively alters the content and dynamics of the condensate.

## Architecture of GABAergic inhibitory synapses

GABA is the major inhibitory neurotransmitter in the brain. Under cryoET, a prominent feature of GABAergic synapses is a uniform thin sheet-like PSD ~12 nm beneath the postsynaptic membrane (Figure 3a and c) [8••]. This thin sheet PSD layer has not been consistently observed with classic EM methods, possibly due to structural deformation caused by dehydration and/or stain accumulation in-between the PSD layer and the membrane. The PSD likely contains gephyrin, the major scaffolding protein in inhibitory synapses, and perhaps other associated molecules [57,58]. Gephyrin contains a G domain, an E domain, and a flexible linker region in between [58]. Structural and biochemical data showed that the gephyrin E domain can form dimers, while the G domain can form trimers, leading to a hypothetical architecture of regular hexagonal lattices [59,60•]. However, such lattices have not been observed under EM [8••,^61^], perhaps because the resolution is not sufficient to identify such fine structures. Alternatively, it is possible that gephyrin molecules do not form rigid lattices because of intrinsic flexibility of the linker, or low binding affinity among the E or G domains [60•]. Indeed, *in vitro* study showed that gephyrin could form an amorphous meshwork [60•]. Further *in situ* studies with high-resolution cryoET may reveal a more precise picture of gephyrin organization in inhibitory synapses.

Also with cryoET, densities that have shapes similar to the atomic structure of GABA_A_R have been identified in inhibitory synapses of cultured hippocampal neurons (Figure 3a and d). On the cytoplasmic side, a putative GABA_A_R particle often contained a thin ‘neck-like’ structure, which in turn often links with a globular ‘head’ (Figure 3b). The neck could be the cytoplasmic domain of GABA_A_R, and the globular head could be gephyrin or the dimer of gephyrin E domain, as the cytoplasmic domain of GABA_A_R is known to directly interact with the gephyrin E domain [62]. It is possible that the neck density is also contributed by collybistin, which could interact with both gephyrin and the cytoplasmic domain of GABA_A_R [63], and plays a critical role for inhibitory PSD organization [64]. Additionally, some densities similar to the neck structure were not linked to the GABA_A_R density but directly projected from the membrane (unpublished observations). We speculate that these densities are collybistin molecules that bind to the PI3P lipid on membrane [65]. Additionally, a filamentous network, possibly composed of scaffolding or signaling proteins, extending ~100 nm from gephyrin layer into the cytoplasm was also observed [61]. These heterogeneous interactions onto the gephyrin layer suggest that inhibitory synapses utilize a gephyrin framework that accommodates multiple interfacing proteins such as GABA_A_R, collybistin and other proteins, ensuring the functioning of inhibitory synapses.

## Future perspective

Technological advances in electron microscopy in the past decades have opened a new door to directly observe molecular organization *in situ*, and promise new conceptual breakthroughs in the future. In particular, high resolution cryoET is able to identify and locate individual protein complexes. For example, individual hexamers and pentamers of the HIV capsid protein were mapped to the tomograms on conical HIV capsid [66]. Moreover, proteins in different conformational states or interacting with different partners can be determined through 3D classification. With the help of phase plate and 3D classification, substrate processing and ground states of proteasomes were identified in the neuronal processes, providing an *in situ* molecular census for the proteasome [67••]. It is possible that with further improved resolution, and perhaps with the help of specific nanobodies, one might even be able to identify different subtypes of neurotransmitter receptors that have unique functional properties *in situ*. Identification of these proteins and complexes would elucidate the organizational principles and interaction networks underlying synaptic development and plasticity with single molecule precision, rather than studying the average behaviors of protein ensembles.

Synapses display a remarkable heterogeneity in their functional properties [68,69]. To correlate such functional heterogeneity with the structural features of individual synapses revealed by electron microscopy, an effective approach is to use CLEM to acquire the cryo tomograms guided by fluorescence labeling of individual synapses in either live or frozen-hydrated state. As a proof of concept, inhibitory and excitatory synapses were identified and analyzed by cryoCLEM [8••]. Conceivably, synapses in different signaling or plasticity states labeled by specific fluorescent protein markers can be located by fluorescence microscopy and studied by cryoET. Furthermore, subsynaptic localization of specific proteins may be imaged by supersolution optical microscopy [3•,^32^,^49^]. Correlating such localization information with cryoET would facilitate resolving the structure and organization of identified synaptic proteins and their interacting complexes, especially when the proteins are too small to be identified by cryoET alone.

Synapses are highly dynamic devices, inside which molecules and organelles move constantly to carry out synaptic functions [68,69]. Electron microscopy can only provide a collection of ‘snapshots’ of different synapses in action. To understand the dynamic process inside synapses, time-resolved electron microscopy is a promising strategy to synchronize synapse in different dynamic states [70]. For example, by controlling the timing of optogenetic stimulation before high-pressure freezing, ultrafast endocytosis was discovered to take place 100–200 ms after action potential firing [71]. A similar approach with even high temporal resolution can be developed for cryoET, in which neuronal synapses can be stimulated at different time points, by either electrical, chemical or optical means, before plunge freezing. These tools will enable studying different phases of synaptic transmission, development and plasticity, ultimately leading to a 4-D picture of synapses in unprecedented details.
